# An Exploratory Trial of EPI-589 in Amyotrophic Lateral Sclerosis (EPIC-ALS): Protocol for a Multicenter, Open-Labeled, 24-Week, Single-Group Study

**DOI:** 10.2196/42032

**Published:** 2023-01-30

**Authors:** Shotaro Haji, Koji Fujita, Ryosuke Oki, Yusuke Osaki, Ryosuke Miyamoto, Hiroyuki Morino, Seiichi Nagano, Naoki Atsuta, Yuki Kanazawa, Yuki Matsumoto, Atsuko Arisawa, Hisashi Kawai, Yasutaka Sato, Satoshi Sakaguchi, Kenta Yagi, Tatsuto Hamatani, Tatsuo Kagimura, Hiroaki Yanagawa, Hideki Mochizuki, Manabu Doyu, Gen Sobue, Masafumi Harada, Yuishin Izumi

**Affiliations:** 1 Department of Neurology Tokushima University Graduate School of Biomedical Sciences Tokushima Japan; 2 Department of Medical Genetics Tokushima University Graduate School of Biomedical Sciences Tokushima Japan; 3 Department of Neurotherapeutics Osaka University Graduate School of Medicine Suita Japan; 4 Department of Neurology Osaka University Graduate School of Medicine Suita Japan; 5 Department of Neurology Aichi Medical University School of Medicine Nagakute Japan; 6 Department of Biomedical Information Sciences Tokushima University Graduate School of Biomedical Sciences Tokushima Japan; 7 Department of Radiology Tokushima University Graduate School of Biomedical Sciences Tokushima Japan; 8 Department of Diagnostic and Interventional Radiology Osaka University Graduate School of Medicine Suita Japan; 9 Department of Radiology Aichi Medical University School of Medicine Nagakute Japan; 10 Clinical Research Center for Developmental Therapeutics Tokushima University Hospital Tokushima Japan; 11 Sumitomo Pharma Co, Ltd Osaka Japan; 12 The Translational Research Center for Medical Innovation Foundation for Biomedical Research and Innovation at Kobe Kobe Japan; 13 Aichi Medical University School of Medicine Nagakute Japan

**Keywords:** amyotrophic lateral sclerosis, biomarker, clinical trial, magnetic resonance imaging, oxidative stress

## Abstract

**Background:**

Amyotrophic lateral sclerosis (ALS) is a fatal neurodegenerative disorder, with its currently approved drugs, including riluzole and edaravone, showing limited therapeutic effects. Therefore, safe and effective drugs are urgently necessary. EPI-589 is an orally available, small-molecule, novel redox-active agent characterized by highly potent protective effects against oxidative stress with high blood-brain barrier permeability. Given the apparent oxidative stress and mitochondrial dysfunction involvement in the pathogenesis of ALS, EPI-589 may hold promise as a therapeutic agent.

**Objective:**

This protocol aims to describe the design and rationale for the EPI-589 Early Phase 2 Investigator-Initiated Clinical Trial for ALS (EPIC-ALS).

**Methods:**

EPIC-ALS is an explorative, open-labeled, single-arm trial that evaluates the safety and tolerability of EPI-589 in patients with ALS. This trial consists of 12-week run-in, 24-week treatment, and 4-week follow-up periods. Patients will receive 500 mg of EPI-589 3 times daily over the 24-week treatment period. Clinical assessments include the mean monthly change of Amyotrophic Lateral Sclerosis Functional Rating Scale–Revised total score. The biomarkers are selected to analyze the effect on oxidative stress and neuronal damage. The plasma biomarkers are 8-hydroxy-2′-deoxyguanosine (8-OHdG), 3-nitrotyrosine (3-NT), neurofilament light chain (NfL), phosphorylated neurofilament heavy chain (pNfH), homocysteine, and creatinine. The cerebrospinal fluid biomarkers are 8-OHdG, 3-NT, NfL, pNfH, and ornithine. The magnetic resonance biomarkers are fractional anisotropy in the corticospinal tract and N-acetylaspartate in the primary motor area.

**Results:**

This trial began data collection in September 2021 and is expected to be completed in October 2023.

**Conclusions:**

This study can provide useful data to understand the characteristics of EPI-589.

**Trial Registration:**

Japan Primary Registries Network jRCT2061210031; tinyurl.com/2p84emu6

**International Registered Report Identifier (IRRID):**

DERR1-10.2196/42032

## Introduction

Amyotrophic lateral sclerosis (ALS) is a fatal neurodegenerative disease that is characterized by progressive upper and lower motor neuronal dysfunction and loss [1]. The mean survival time is 3-5 years after the onset [2]. ALS is diagnosed in 1-2 individuals per 100,000 each year in most countries, whereas its prevalence is approximately 5 cases per 100,000 people, reflecting the rapid disease lethality [3]. The pathogenic processes underlying ALS are considered multifactorial, including complex interaction of genetic factors, oxidative stress, excitotoxicity, and protein aggregation, as well as impaired RNA processing, axonal transport, and mitochondrial function [4]. Thus far, only a few interventional drugs, including the antiglutamatergic small molecule riluzole [5] and the antioxidant edaravone [6], have been approved by the United States Food and Drug Administration for ALS. Riluzole prolongs survival by only 2-3 months [5]. Edaravone, a strong antioxidant and free radical scavenger [7], slows down the loss of motor function in patients with ALS by 33% compared with a placebo [6,8]. A retrospective study demonstrated that edaravone prolonged tracheostomy-free survival [9], and a 5‐year prospective observational study is ongoing to investigate the long-term safety and effects of edaravone on ALS [10,11]. In addition, a cohort study, using propensity score matching, found safety but not disease-modifying benefit of long-term intravenous edaravone for patients with ALS [12]. Considering these circumstances, new therapies for ALS are highly anticipated.

A redox homeostasis dysfunction that causes oxidative stress has been linked to ALS pathogenesis [13], and markers of oxidative stress, such as 3-nitrotyrosine (3-NT) [14,15] and 8-hydroxy-2′-deoxyguanosine (8-OHdG) [16], are reportedly upregulated in the tissue of patients with ALS [17]. As the disease progresses, nutritional deficiency, cachexia, psychological stress, and impending respiratory failure become evident in ALS, which may be associated with a further increased oxidative stress [18]. EPI-589, also known as (R)-troloxamide quinone, is an orally available, blood-brain barrier–penetrable small-molecule designed as a novel redox-active agent to facilitate catalytic activity on oxidative stress control and mitochondrial energy metabolism. EPI-589 was more potent than edaravone in protecting the cells in an oxidative stress and mitochondrial dysfunction model [19,20]. In wobbler mice experiments, EPI-589 revealed dose-dependent effects to slow motor function deterioration [20,21]. Moreover, nonclinical study results revealed that EPI-589 had effects on 8-OHdG and neurofilament [20]. Given the apparent oxidative stress and mitochondrial dysfunction involvement in ALS pathogenesis, EPI-589 may hold promise as a potential therapeutic agent. In a phase 1 study, EPI-589 at 1500 mg per day was well-tolerated [22]. An early phase 2 trial (ClinicalTrials.gov identifier NCT02460679) revealed that EPI-589 at 500 mg twice daily was safe and well-tolerated with effects on disease progression in patients with ALS. However, the modes of action of EPI-589 for ALS remain unclear, and thus, further analysis is needed to elucidate its effects. Herein, we planned an early phase 2, single-arm, open-labeled exploratory trial of EPI-589 (EPI-589 Early Phase 2 Investigator-Initiated Clinical Trial for ALS [EPIC-ALS]). The primary objective of EPIC-ALS is to evaluate the safety and tolerability of EPI-589 at 500 mg 3 times daily for patients with ALS. The exploratory objectives are to monitor clinical course when receiving EPI-589 and to explore plasma, cerebrospinal fluid (CSF), and magnetic resonance imaging (MRI) results for potential EPI-589 biomarkers. An important characteristic of EPIC-ALS is the evaluation of multiple biomarker measurements in parallel with clinical assessment before, during, and after the intervention in patients with ALS. Here we provide an outline of the clinical trial protocol of EPIC-ALS version 1.0, which was prepared on July 29, 2021.

## Methods

### Study Setting

This investigator-initiated, open-labeled, multicenter, single-arm, and early phase 2 exploratory trial is designed to assess the safety and tolerability. Preliminary efficacy including clinical assessments and biomarkers of EPI-589 will be evaluated in patients with sporadic ALS. Patients will be recruited from the following ALS centers in Japan: Tokushima University Hospital, Aichi Medical University Hospital, and Osaka University Hospital. The total trial duration for each patient is 40 weeks, composed of 12-week run-in, 24-week treatment, and 4-week follow-up periods.

### Eligibility Criteria

Patients’ inclusion and exclusion criteria are depicted in Textbox 1. Patients will be assessed for the inclusion criteria 1-9, 11, and 12 at screening, 10 and 11 at the run-in period enrollment, and 11 again at the treatment period enrollment. Patients will need to meet none of the exclusion criteria at screening, run-in, and treatment period enrollment.

**Inclusion/exclusion criteria.**

*Inclusion criteria*

1. Patient providing written informed consent to participate in this trial. (If the patient is underage at the time of consent, written consent from the patient’s legal representative is required in addition to the written consent from the patient.)
2. Patient between 18 and 79 years of age (inclusive) at the time of consent.
3. Patient diagnosed with clinically definite, probable, or probable laboratory-supported sporadic amyotrophic lateral sclerosis (ALS) by the revised El Escorial criteria.
4. Onset of ALS within 1.5 years at screening.
5. Patient with mean monthly progression rate (calculated as follows: mean progression rate = [score at screening − score at onset]/time from onset to screening [expressed as the number of months rounded to one decimal place]) faster than −0.3 calculated based on Amyotrophic Lateral Sclerosis Functional Rating Scale–Revised (ALSFRS-R) total scores during the period from onset to screening (score at onset 48).
6. Patient whose score for each of the first 9 questions of ALSFRS-R is ≥2 points at screening.
7. Patient whose score for each of the 3 respiration-related questions (dyspnea, orthopnea, and breathing insufficiency) of ALSFRS-R is 4 points at screening.
8. Patient whose score for the swallowing-related question of ALSFRS-R is ≥3 points at screening.
9. Patient whose % slow vital capacity is ≥80% at screening. (A predicted value for % slow vital capacity was calculated using the equation of Baldwin et al [23].)
10. Patient on riluzole must be on a stable dose and regimen for at least 30 days prior to enrollment in the run-in period.
11. Patient who is able to visit the trial site as an outpatient.
12. Patient who agrees to provide blood sample to be used for genetic analysis.

*Exclusion criteria*

1. Patient who has concurrent or a history of clinically significant severe disease besides ALS and who is considered by the principal investigator or subinvestigator ineligible for the trial.
2. Patient who has concurrent psychiatric disorder, cognitive disorder, or Parkinson disease.
3. Patient who has undergone tracheostomy.
4. Patient who has used a noninvasive respiratory support device.
5. Patient who has a history of or concurrent drug allergy or severe allergic disease (eg, anaphylactic shock).
6. Patient with concurrent or a history (≤5 years before informed consent) of malignant tumor.
7. Patient from whom cerebral spinal fluid cannot be collected.
8. Patient who is unable to undergo magnetic resonance imaging (including diffusion tensor imaging and magnetic resonance spectroscopy).
9. Patient whose aspartate aminotransferase and alanine aminotransferase levels at screening are ≥3 times higher than the upper limit of normal.
10. Patient whose creatinine kinase at screening is ≥2.5 times higher than the upper limit of normal.
11. Patient whose estimated glomerular filtration rate at screening is <45 mL/min/1.73 m^2^.
12. Patient who is or who may be pregnant or who is breastfeeding.
13. Patient who or whose partner wishes to be pregnant or who does not agree to use adequate contraceptive methods during the period from informed consent to 30 days after the final dose of the investigational drug.
14. Patient who has used edaravone within 30 days before the enrollment in the run-in period.
15. Patient who has changed the dosing regimen of riluzole or who has discontinued the use of riluzole later than the date of informed consent.
16. Patient who has previously received EPI-589.
17. Patient who participated in another clinical trial before the provision of consent for this trial and received another investigational drug within 30 days before consent for this trial (or within 5 times the half-life of another investigational drug if 5 times the half-life of another investigational drug is longer than 30 days) or patient who is participating or scheduled to participate in another clinical trial at the time of consent for the present trial.
18. Patient who has received cell therapy or gene therapy before provision of informed consent.
19. Patient who is, in the opinion of the principal investigator or subinvestigator, unsuitable to participate in this trial.

### Interventions

Patients will be administered EPI-589 at 500 mg (2 tablets of 250 mg) 3 times daily at least 60 minutes before meals during the 24-week treatment period. Based on the previous phase 1 results [22], when administered under fed conditions, the C_max_ decreased to 62.6% of the C_max_ under fasting conditions. Area under the curve was comparable between the fasted and fed states. Therefore, the timing of administration was decided “before meals” to maximize the exposure. Sumitomo Pharma Co., Ltd. will provide EPI-589 free of charge. Eligible patients should be on a stable dose of riluzole. Patients will be prohibited from the concomitant use of drugs with antioxidant potential, including edaravone, coenzyme Q10, and minocycline, as well as other potentially effective ALS drugs and therapies to evaluate the effects of EPI-589 on oxidative stress (Multimedia Appendix 1).

### Outcomes, Patient Timeline, and Data Management

Trial assessments and procedures per visit are presented in Multimedia Appendix 2. Genetic analysis, including C9orf72 repeat expansion and whole-exome sequence, will be performed to avoid confounding due to genetic effects. Genetic analysis for C9orf72 repeat expansion will be evaluated at Tokushima University, whereas the whole-exome sequencing will be conducted at TAKARA Bio Inc.

The clinical course will be assessed by the change in Amyotrophic Lateral Sclerosis Functional Rating Scale–Revised (ALSFRS-R) scores, as well as by the mean change rate of ALSFRS-R scores per month, time to death, tracheostomy, whole-day use of noninvasive positive pressure ventilation, and physical findings. Variations in ALSFRS-R scores between assessors may be minimized by having each assessor master the scale in advance through a training program. Physical findings include slow vital capacity, manual muscle test total score, grip strength, the modified Norris scale total score, and the Amyotrophic Lateral Sclerosis Assessment Questionnaire-40 (ALSAQ-40) total score.

The safety will be evaluated by adverse events, laboratory tests, vital signs, 12-lead electrocardiogram, and the Columbia-Suicide Severity Rating Scale. To rule out any contraindication for receiving EPI-589, laboratory tests will be performed by a local laboratory, along with urinalysis and urine pregnancy tests.

The biomarkers in plasma and CSF were set to analyze the effect on oxidative stress (8-OHdG and 3-NT), neuronal damage (neurofilament light chain [NfL] and phosphorylated neurofilament heavy chain [pNfH]), and others such as ornithine, creatinine, and homocysteine. Plasma biomarkers are 8-OHdG, 3-NT, NfL, pNfH, homocysteine, and creatinine. CSF biomarkers are 8-OHdG, 3-NT, NfL, pNfH, and ornithine. Plasma and CSF samples will be immediately centrifuged after collection at 3000 rpm (1750*g*) for 10 minutes at 4℃ and stored at −80℃ at each site. The samples will be gathered and analyzed at a laboratory center.

The MRI data will be obtained with a 3.0-Tesla scanner at each site (Tokushima University Hospital and Osaka University Hospital, GE Healthcare; Aichi Medical University Hospital, Siemens Healthineers). The sequences will consist of T2-weighted imaging, 3D T1-weighted imaging, diffusion tensor imaging (30 motion probing gradient directions, b=1000 s/mm^2^), and magnetic resonance spectroscopy (MRS) obtained with point-resolved spectroscopy with short and long echo time (30 and 135 ms, respectively) in the bilateral primary motor area, with a total scan time of less than 30 minutes for each patient. Optional sequences will include synthetic MRI with magnetic resonance image compilation and chemical exchange saturation transfer echo-planar imaging in the primary motor area. The imaging acquisition parameters were standardized beforehand (Multimedia Appendix 3). Anonymized MRI data will be sent to the Imaging Review Committee at Tokushima University. MRI biomarkers are diffusion tensor imaging–derived fractional anisotropy and apparent diffusion coefficient in the corticospinal tract; cerebral cortex parameters derived from 3D T1-weighted imaging; and *N*-acetylaspartate adjusted by creatine, choline, or creatine + choline measured by MRS in the primary motor area [24]. MRI biomarker values will be harmonized across the 3 institutions using healthy control MRI data obtained in an ancillary multicenter study.

Data will be abstracted into standardized data collection forms at patient enrollment. The ALSFRS-R will be assessed every 4 weeks through this trial. Clinical parameter assessments, including the manual muscle test, grip strength, modified Norris scale, and ALSAQ-40, are set to be evaluated at visits 2, 3, 6, and 9, where plasma biomarkers at visits 2, 3, 6, 9, and 10; CSF and MRI biomarkers at visits 2, 3, and 9; and optional MRI biomarkers at visits 6 and 10 will be collected and evaluated. Data will be monitored and audited by an independent contract research organization (CMIC Co., Ltd.).

Severe adverse events (SAEs) will be reported by the investigator within 24 hours of awareness by telephone, fax, email, or written document. As the secondary reporting, “SAEs reporting” will be submitted within 7 days of the investigator’s awareness if detailed information is not included in the primary reporting. However, in case of an adverse reaction that should be reported to the regulatory authority within 7 days, care should be taken to obtain information within the period for reporting to the regulatory authority. The Safety Monitoring Board will review data on SAEs. The Safety Monitoring Board is an organization independent of the trial representative, coordinating investigator, investigators, subinvestigators, or contract research organization, and has authority to recommend the discontinuation of the study if there is any safety concern. The investigator, after consultation with the coordinating investigator, will seek advice from the Safety Monitoring Board about the continuation or discontinuation of the study as needed and determine measures to be taken. If an SAE (eg, death or a life-threatening event) is observed, for which a causal relationship with the investigational drug cannot be ruled out, enrollment will be suspended and a review of the Safety Monitoring Board will be sought.

The coordinating investigator will terminate or interrupt the entire trial in the following cases: (1) death or a life-threatening SAE, for which a relationship with the investigational drug cannot be ruled out, occurs (patient enrollment should be suspended in this case); (2) following the Safety Monitoring Board’s recommendation on the termination or interruption of the entire trial, the coordinating investigator judges that the entire trial should be terminated or interrupted; (3) the Institutional Review Board recommends that the entire trial should be terminated or interrupted; (4) a change is made to the development policy of the coordinating investigator; (5) the regulatory authority recommends the termination of the trial; (6) any other situations, where partial or complete termination or interruption of the trial is needed, occur.

### Telephone Survey

Telephone-based assessment will be permitted for the evaluation of adherence, ALSFRS-R score, events, tracheostomy, concomitant medications and therapies, and adverse events at visits TEL1, TEL2, 5, 7, or 8 because the ALSFRS-R telephone-based assessment was shown as reliable as in-person visits [25,26]. However, the telephone-based assessment will be used only in a state of emergency, including the COVID-19 pandemic, other infection pandemics, natural disaster, and terrorism, to ensure patient safety. Consecutive telephone-based assessment is prohibited.

### Sample Size and Recruitment

Based on the results of the previous early phase 2 trial (NCT02460679), and also in light of the feasibility of this study, 10 patients were selected as the target sample size. Information on this trial will be disseminated via press coverage and websites to achieve timely adequate patient enrollment.

### Statistical Analysis

The primary analysis, based on intention-to-treat principles, includes the full analysis set (FAS) including patients who received at least one dose of the investigational product and had a baseline measurement and at least one postbaseline measurement of efficacy endpoints or biomarkers, regardless of any protocol deviation. The secondary analysis includes the per-protocol set excluding patients with medication compliance of less than 70%, patients whose dosing period was less than 8 weeks, and patients with an important protocol deviation from FAS. The analysis with the per-protocol set is performed as a sensitivity analysis that confirms the consistency of FAS results. All patients who received EPI-589 are included in the safety analysis.

Changes of 20%-25% or greater in the ALSFRS-R slope would be a clinically meaningful effect [27]. Based on the finding, responders are defined as patients with an improvement in the rate of declined ALSFRS-R total score by 30% compared with the rate in the run-in period. The detailed definition of responders is presented in the following section.

[The mean change of ALSFRS-R total score per month in the treatment period (each visit) – The mean change of ALSFRS-R total score per month in the run-in period]/|The mean change per month of ALSFRS-R total score in the run-in period| × 100 ≥30%.

The proportion of responder and CI will be calculated for each visit. In addition, a Z-test will be performed for the binomial proportion assuming a 10% threshold for the proportion of responders. However, patients with no change or increase in the ALSFRS-R total score in the run-in period will be excluded from this analysis. A total of 10 patients with a 2-sided α of .1 will provide more than 80% power to reject the null hypothesis (the proportion of responders equals 10%) based on a previous clinical trial (NCT02460679).

The mean ALSFRS-R changes per month will be compared between the run-in and treatment periods using the paired *t* test. In addition, the mixed model will be used to compare the ALSFRS-R slope between the run-in and treatment periods. Some evidence suggested that functional decline may not be linear [28-30]; however, a previous open-label 24-week extension study revealed that ALSFRS-R scores changed almost linearly from baseline through 48 weeks in the edaravone treatment group [8]. The change from baseline and the change per month for all continuous efficacy endpoints will be descriptively summarized at each visit and compared between the run-in and treatment periods using the paired *t* test. The number and percentage of patients with each outcome will be summarized by visit for parameters with categorical outcomes. Time to event since the first dose of the study drug will be analyzed by the Kaplan-Meier method and plotted by the Kaplan-Meier curve. The statistical significance will be set at a 2-sided level of .1. All analysis will be conducted using SAS software (version 9.4 or higher; SAS Institute Inc.).

We will also provide a list of adverse events, the number of patients completing the study, the CONSORT (Consolidated Standards of Reporting Trials) flow diagram, and the mean changes from baseline in the biomarkers. Individual data trajectories in a panel plot will also be made.

### Patient and Public Involvement

Patients or the public were not involved in the design, conduct, reporting, or dissemination plans of our research.

### Ethics Approval and Dissemination

This trial was approved by the Institutional Review Board of Tokushima University Hospital in August 2021 (approval number 2125 [03-10]), Aichi Medical University Hospital in October 2021 (approval number 202115), and Osaka University Hospital in November 2021 (approval number 219005-A) based on the Declaration of Helsinki, Good Clinical Practice, and associated Japanese regulations. The protocol has been approved by the Japanese regulatory authority Pharmaceuticals and Medical Devices Agency before the commencement of the trial. This trial was registered at the Japan Registry of Clinical Trials (JRCT ID: jRCT2061210031; registered on August 27, 2021 and last modified on July 25, 2022). Substantial amendments of the protocol must be approved by each institutional review board. Written informed consent will be obtained from all potentially eligible patients after the investigators provide a detailed explanation. Personal information about potential and enrolled patients will be masked to protect confidentiality before, during, and after the trial. If health injury (eg, adverse drug reaction) occurs in the patient in association with this trial, the head of the trial site will follow necessary procedures including provision of adequate medical care to the patient. For death/physical impediment that is within the scope of compensation, compensation will be paid in accordance with the terms and conditions of a clinical trial insurance contract. Compensation will not be paid if the health injury is not related to the trial or is caused by the patient’s intentional misconduct or gross negligence or the protocol treatment has no beneficial effects. This trial received funding from Sumitomo Pharma Co., Ltd.

## Results

This trial began data collection in September 2021 and is expected to be completed in October 2023. The ancillary study to obtain healthy control MRI data has been approved by the Ethics Committee of Tokushima University Hospital (approved on May 31, 2021; No. 3997), the Aichi Medical University School of Medicine Ethical Review Board, and the Osaka University Research Ethics Committee. The principal and coordinating investigators (YI and KF) will have access to the final trial data set. The results will be disseminated by presentations at scientific meetings and published in a peer-reviewed scientific journal.

## Discussion

The pathogenic processes that underlie ALS remain unclear; however, accumulating evidence suggests that oxidative stress and mitochondrial dysfunction may play a pivotal role [4]. Oxidative stress is typically defined as an imbalanced production of free oxygen radicals, reactive oxygen species (ROS), or reactive nitrogen species. The mitochondrial wall contains the respiratory chain, which consumes 85%-90% of the total oxygen that is consumed by the body, but also produces the highest ROS levels in the cell due to electrons that escape from the respiratory chain and oxygen molecules that are incompletely reduced in aerobic metabolism [1]. Neurons are particularly susceptible to damage from oxidative stress for the following reasons [31]. First, neurons are remarkably large cells with large metabolic and high oxygen demand causing increased ROS production [32-34]. Second, motor neurons express relatively low calcium-binding protein levels. Thus, excess calcium ions tend to enter the mitochondria, leading to increased ROS production [35,36]. Third, some protective antioxidant protein expression is lower in motor neurons than in other cell types [37,38]. Patients with familial and sporadic ALS have elevated oxidative damage biomarkers in the spinal cord and motor cortex, mostly in large ventral horn motor neurons [14,39-41].

Edaravone is an antioxidant drug that is approved for ALS, which improves motor functions or oxidative stress in mutant SOD1 transgenic mice and rats and patients with ALS [6,42-46]. Edaravone reacts with oxidative radicals by an electron transfer reaction that consumes edaravone anion, which is converted to edaravone radical. Edaravone radical is transformed into 4,5-dione via edaravone peroxyl radicals with molecular oxygen reaction, followed by hydrolysis, to afford 2-oxo-3-(phenylhydrazone)-butanoic acid [47]. Edaravone increases nuclear factor erythroid 2–related factor 2 (Nrf2) expression, an oxidative stress inhibitor [48]. The overexpression of Nrf2 in astrocytes significantly delayed the onset and extended the survival in the SOD1-G93A mouse model of ALS [49]. Nrf2 overexpression in astrocytes reversed SOD1-G93A toxicity and protected the motor neurons from neurodegeneration in the SOD1-G93A mouse model [49]. In humans, edaravone significantly reduced the oxidative stress biomarkers, including 3-NT, in CSF of patients with ALS at an early stage [46]. However, edaravone has a limited effect, thus more effective drugs are urgently needed.

EPI-589 offers an effective novel antioxidant for ALS with advantages in drug delivery because it is orally bioavailable and is a small-molecule catalyst with high blood-brain barrier permeability. Previous studies revealed antioxidant reactions of the reduced EPI-589 form in the cell-free system. Moreover, EPI-589 is expected to not be hydrolyzed when scavenging radicals and to preserve the repeated catalytic activity. *In vitro* studies have revealed EPI-589 as more protective of cell viability against oxidative stress than edaravone (approximately 1000 times more potent with a difference in half maximal effective concentration for fibroblast cells derived from patients with ALS with FUS c.1566G>A mutation and SOD1 E50K mutation) [19,20].

A phase 1 trial in healthy participants (jRCT2071210022) [22] revealed that EPI-589 was safe and well-tolerated as a single daily dose of up to 1000 mg. In addition, EPI-589 was safe and well-tolerated when participants received 750 mg 2 times daily (1500 mg per day) in the multiple-dose part. In a phase 2 open-label trial (NCT02460679), 19 patients with ALS received EPI-589 500 mg 2 times daily. As in the phase 1 studies, no EPI-589–related SAEs were observed and most adverse events were mild or moderate. In our trial, we set the dose of EPI-589 to 500 mg 3 times daily because 1500 mg per day is the maximum daily dose used in the previous phase 1 study. Although EPI-589 500 mg 2 times daily showed possible efficacy on ALS progression in a phase 2 open-label trial (NCT02460679), given that the terminal elimination half-life of EPI-589 is relatively short [22], dosing 3 times a day rather than 2 times a day is preferable to maximize the efficacy.

EPI-589 slowed the symptomatic and pathophysiological progression of the wobbler mouse motor neuron disease model, with a higher effect than edaravone or riluzole [20,21,50]. Moreover, the disease-modifying effect was higher in the concomitant use of EPI-589 and riluzole than a single use of EPI-589 [20,50]. Thus, patients with concomitant use of riluzole were considered eligible for this trial.

We planned to monitor potential biomarkers throughout the trial duration to gain insight into the modes of action of EPI-589 in human patients. EPI-589 acts as a redox-active protectant against oxidative stress under experimental conditions relevant to ALS. Therefore, we will analyze the oxidative stress biomarkers (8-OHdG and 3-NT) as well as neuronal damage biomarkers (NfL and pNfH) [51] in plasma and CSF to obtain robust data to support this concept. We will evaluate other relevant factors, including ornithine in CSF and creatinine and homocysteine in plasma. Of these, plasma 3-NT, which reflects peroxynitrite-mediated oxidative damage, has been studied in multiple sclerosis [52] and Parkinson disease [53] but not in ALS. Moreover, prior studies revealed that a wobbler mouse model had a significantly increased plasma pNfH and decreased *N*-acetylaspartate level on MRS in the spinal cord compared with the wild type; EPI-589 administration improved these biomarkers [20]. These results suggest the effect on motor neurodegeneration, thus we set to evaluate *N*-acetylaspartate in the primary motor area. In addition, previous reports showed longitudinal changes in fractional anisotropy in the corticospinal tract that correlated with the slope of ALSFRS-R [54,55]. Therefore, we will assess fractional anisotropy in the corticospinal tract.

This trial has a couple of limitations. First, the trial size is relatively small. Second, this is an open-labeled, single-arm trial. Instead of a placebo, the 12-week run-in period will be used as control. As the primary objective of this trial is to evaluate safety and tolerability, placebo-controlled trials will be needed to confirm the efficacy.

In summary, this trial will evaluate the safety and tolerability of EPI-589 in combination with longitudinal changes in clinical parameters and biomarker candidates of plasma, CSF, and MRI. Data of the multiple biomarkers will help assess the modes of action of EPI-589 on ALS. This pilot trial will provide insights into the variability of efficacy endpoints and the feasibility of the next large randomized controlled trial.

## Appendix

Multimedia Appendix 1 List of prohibited drugs and therapies.

Multimedia Appendix 2 Schedule of assessments.

Multimedia Appendix 3 Protocols of magnetic resonance imaging and spectroscopy.

## Abbreviations

3-NT: 3-nitrotyrosine
8-OHdG: 8-hydroxy-2′-deoxyguanosine
ALS: amyotrophic lateral sclerosis
ALSAQ-40: Amyotrophic Lateral Sclerosis Assessment Questionnaire-40
ALSFRS-R: Amyotrophic Lateral Sclerosis Functional Rating Scale–Revised
CONSORT: Consolidated Standards of Reporting Trials
CSF: cerebrospinal fluid
EPIC-ALS: EPI-589 Early Phase 2 Investigator-Initiated Clinical Trial for ALS
FAS: full analysis set
MRI: magnetic resonance imaging
MRS: magnetic resonance spectroscopy
NfL: neurofilament light chain
pNfH: phosphorylated neurofilament heavy chain
ROS: reactive oxygen species
SAE: severe adverse event

## Data Availability

Data sharing is not applicable to this article as no data sets were generated or analyzed.
