# Supplementary material for: An Exploratory Trial of EPI-589 in Amyotrophic Lateral Sclerosis (EPIC-ALS): Protocol for a Multicenter, Open-Labeled, 24-Week, Single-Group Study
Source: JMIR Res Protoc. 2023 Jan 30;12:e42032. doi: 10.2196/42032 (PMC9926342; doi:10.2196/42032)
Supplement: Multimedia Appendix 1 [file resprot_v12i1e42032_app1.pdf]

Multimedia Appendix 1. List of prohibited drugs and therapies

---

Drugs

- edaravone
- coenzyme Q10
- minocycline
- insulin-like growth factor 1
- dextromethorphan hydrobromide hydrate and quinidine sulfate
- tamoxifen
- thalidomide
- meloxicam
- arimoclomol
- perampanel
- bosutinib
- ropinirole
- ibudilast
- mecobalamin
- ravulizumab
- eculizumab
- sodium phenylbutyrate
- ursodeoxycholic acid
- memantine
- trazodone

Other treatment

- Wearable Cyborg Hybrid Assistive Limb (HAL)® (CYBERDYNE, INC., Tsukuba, Japan)
- NeurRX Diaphragm Pacing System (DPS)® (Synapse Biomedical, Inc., Engien Les Bains, France)
