# Supplementary material for: An Exploratory Trial of EPI-589 in Amyotrophic Lateral Sclerosis (EPIC-ALS): Protocol for a Multicenter, Open-Labeled, 24-Week, Single-Group Study
Source: JMIR Res Protoc. 2023 Jan 30;12:e42032. doi: 10.2196/42032 (PMC9926342; doi:10.2196/42032)
Supplement: Multimedia Appendix 2 [file resprot_v12i1e42032_app2.pdf]

Multimedia Appendix 2. Schedule of Assessments

Assessment[a)]	Informed consent	Screening	Run-in period (12 weeks)				Treatment period (24 weeks)						Follow-up period (4 weeks)
Visit No.[b)]		Visit 1	Visit 2[j)]	Visit TEL 1 [k)]	Visit TEL 2 [k)]	Visit 3	Visit 4	Visit 5[l)]	Visit 6	Visit 7[l)]	Visit 8[l)]	Visit 9 (or discontinuation[m)])	Visit 10[n)]
Timing		Screening	Run-in period Start day (Pre Day 1)	Run-in period Week 4 (Pre Day 29)	Run-in period Week 8 (Pre Day 57)	Run-in period End of Week 12 (Pre Day 85 / Day 0)	Treatment period Week 4 (Day 28)	Treatment period Week 8 (Day 56)	Treatment period Week 12 (Day 84)	Treatment period Week 16 (Day112)	Treatment period Week 20 (Day140)	Treatment period End of Week 24 (Day 168)	Follow-up period Completion
Time window		–	Visit 1 + ≤15days	±7 days	±7 days	±7 days	±7 days	±7 days	±7 days	±7 days	±7 days	±7 days	Visit 9 + 21 to 35 days
Informed consent[c)]	✓												
Physical examination		✓	✓			✓	✓	✓	✓	✓	✓	✓	✓
Confirmation of trial continuation/completion			✓	✓	✓	✓	✓	✓	✓	✓	✓	✓	✓
Demographics		✓											
Eligibility confirmation		✓	✓			✓							
Enrollment			✓			✓							
Investigational drug prescription						✓	✓	✓	✓	✓	✓		
Confirmation of treatment compliance							✓	✓	✓	✓	✓	✓	
Vital signs (blood pressure, pulse rate, body temperature)		✓	✓			✓	✓	✓	✓	✓	✓	✓	✓
12-lead ECG		✓	✓			✓	✓		✓			✓	✓
Weight		✓	✓			✓	✓	✓	✓	✓	✓	✓	✓
Height		✓											
Pregnancy test[d)]		✓										✓	
ALSFRS-R		✓	✓	✓	✓	✓	✓	✓	✓	✓	✓	✓	✓
%SVC		✓	✓			✓			✓			✓	✓
MMT, grip strength, Modified Norris scale, ALSAQ-40			✓			✓			✓			✓	
Occurrence of events[e)]			✓			✓	✓	✓	✓	✓	✓	✓	✓
CSF sampling[f)]			✓			✓						✓	
MRI[g)]			✓			✓			✓			✓	✓
Plasma biomarker measurement			✓			✓			✓			✓	✓
CSF biomarker measurement			✓			✓						✓	
C-SSRS[h)]						✓	✓		✓			✓	✓
Laboratory tests (blood, urine)[i)]		✓	✓			✓	✓	✓	✓	✓	✓	✓	✓
Sampling for genetic analysis		✓											
Confirmation of tracheotomy status			✓	✓	✓	✓	✓	✓	✓	✓	✓	✓	✓
Confirmation of concomitant medications/therapies		✓	✓	✓	✓	✓	✓	✓	✓	✓	✓	✓	✓
Adverse events	←	—	—	—	—	—	—	—	—	—	—	—	→

Visit 2 (at the start of the run-in period) is regarded as Pre Day 1. Administration of the investigational drug will be started the next day of Visit 3, and the start day of trial treatment is regarded as Day 1.

a) Plasma-based biomarkers are set to be evaluated at the start of the run-in period, the end of the run-in period, 12 weeks of the treatment period, the end of the treatment period, and the end of the follow-up period. CSF- and MRI-based biomarkers are set to be evaluated at the start of the run-in period, the end of the run-in period, and the end of the

treatment period. If possible, MRI-based biomarkers will be evaluated at 12 weeks of the treatment period and the end of the follow-up period.

b) Visit 2 (at the start of the run-in period) is regarded as preday 1. Administration of the investigational drug will be started the next day of visit 3, and the start day of trial treatment is regarded as day 1.

c) Consent will be obtained in writing prior to implementation of any procedure needed for the trial, discontinuation of prohibited concomitant medications or conduct of examinations.

d) To be performed only in women of childbearing potential before menopause.

e) An event is defined as whole-day ($\geq$ 22 hours) use of noninvasive respiratory support, use of invasive respiratory support, or death.

f) Hospital admission may be permitted for CSF sampling.

g) MRI at Week 12 of the treatment period and at completion of the follow-up period is optional and will be performed wherever possible.

h) The C-SSRS Baseline version will be used at the visit at completion of the run-in period and the C-SSRS Since Last Visit version will be used at the subsequent visits.

i) Urinary human chorionic gonadotropin quantitative test will be performed at screening and Week 24 of the treatment period (or day of discontinuation).

j) Assessments at Visit 2 (start of the run-in period) may be performed at Visit 1. If assessments at Visit 2 are performed within 7 days of Visit 1 (including the day of Visit 1), data from Visit 1 may be used for vital signs (blood pressure, pulse rate, body temperature), body weight, 12-lead ECG, ALSFRS-R, %SVC, and laboratory tests, instead of examining these items at Visit 2. In such a case, data from Visit 1 will be cited in the case report form (on the page for Visit 2).

k) Confirmation of continued trial participation and ALSFRS-R assessment at Weeks 4 and 8 of the run-in period will be performed via telephone, in principle. However, they may be performed through the patient's trial site visits.

l) For Weeks 8, 16, and 20 of the treatment period, if the patient cannot visit the trial site under the influence of the Coronavirus Disease 2019 pandemic, etc. (including natural disasters, other infectious pandemics, and terror attacks) or for safety reasons, remotely evaluable items (i.e., confirmation of treatment compliance, ALSFRS-R assessment, confirmation of occurrence of events, confirmation of tracheotomy status, confirmation of concomitant medications/therapies, and confirmation of AEs) may be evaluated via telephone and remotely non-evaluable items may be handled as missing data. In the event of compelling circumstances, the investigational drug may be sent to the patient by mail. However, remote assessments for 2 consecutive visits will not be permitted.

m) Examinations at discontinuation will not be performed for patients who have taken no dose of the investigational drug before discontinuation. Examinations at discontinuation will be performed within 14 days of the day of discontinuation (Day 0).

n) Follow-up will also be performed for patients discontinued from the trial wherever possible. However, follow-up will not be performed for patients who have taken no dose of the investigational drug before discontinuation.

Abbreviations: AE, adverse events; ALSAQ-40, Amyotrophic Lateral Sclerosis-Assessment Questionnaire 40; ALSFRS-R, Amyotrophic Lateral Sclerosis Functional Rating Scale–Revised; CSF, cerebrospinal fluids; C-SSRS, Columbia-Suicide Severity Rating Scale; IC, informed consent; MMT, manual muscle testing; MRI, magnetic resonance imaging; SAE, serious adverse events; SVC, slow vital capacity.
