# Supplementary material for: An Exploratory Trial of EPI-589 in Amyotrophic Lateral Sclerosis (EPIC-ALS): Protocol for a Multicenter, Open-Labeled, 24-Week, Single-Group Study
Source: JMIR Res Protoc. 2023 Jan 30;12:e42032. doi: 10.2196/42032 (PMC9926342; doi:10.2196/42032)
Supplement: Multimedia Appendix 3 [file resprot_v12i1e42032_app3.pdf]

Multimedia Appendix 3. Protocols of magnetic resonance imaging and spectroscopy

Abbreviations: AP, anterior-posterior; ASSET, array spatial sensitivity encoding technique; CHESS, chemical shift selective; CSI, chemical shift imaging; FH, foot-head; FOV, field of view; FSE, fast spin echo; LR, left-right; MAGiC, magnetic resonance image compilation; MPG, motion probing gradient; MPRAGE, magnetization prepared rapid acquisition with gradient echo; MRS, magnetic resonance spectroscopy; NEX, number of excitations; NSA, number of signal averages; PRESS, point resolved spectroscopy; RL, right-left; SE-EPI, spin-echo echo-planer imaging; SPGR, spoiled gradient echo; SPIR, Spectral inversion recovery; TE, echo time; TR, repetition time; WFS, water fat shift.

T2-weighted imaging

Head coil		12ch head coil
Pulse sequence		FSE
TR (ms)		3,000 or 5,000
TE (ms)		95–99.2
NEX/NSA		1
Bandwidth (kHz, Hz/Px, WFS(Px))		120 kHz
Matrix size (reconstruction)		512*512
Matrix size (acquisition)		256*256
FOV	FH (mm)	144
	AP (mm)	220
	RL (mm)	220
Voxel size	FH (mm)	6
	AP (mm)	0.57
	RL (mm)	0.86
Slice	Thickness (mm)	5
	Interslice spacing (mm)	1
	Number of slices	25
	Slice orientation	Axial
Phase encoding direction		LR
Shim (auto/manual)		Auto
Scan duration		0:36

3D T1-weighted imaging

Head coil		12ch head coil
Pulse sequence		SPGR or MPRAGE
Acceleration mode		ASSET
Acceleration factor		2
TR (ms)		8 (SPGR), 1,800 (MPRAGE)
TI (ms)		650 (SPGR), 800 (MPRAGE)
TE (ms)		3
Flip angle (deg)		8 (SPGR), 10 (MPRAGE)
Matrix size (reconstruction)		256*256
Matrix size (acquisition)		256*256
FOV	FH (mm)	171
	AP (mm)	240
	RL (mm)	240
Voxel size	FH (mm)	0.9
	AP (mm)	0.9
	RL (mm)	0.9
Slice	Thickness (mm)	0.9
	Interslice spacing (mm)	-
	Number of slices	190
	Slice orientation	Axial
Phase encoding direction		LR
NEX/NSA		1
Bandwidth (kHz, Hz/Px, WFS(Px))		35.71 kHz
Shim (auto/manual)		Auto
Fat suppression (Yes/No)		No
Fat suppression mode (CHESS/SPIR/Water excitation)		No
Scan duration		5:56

Diffusion tensor imaging

Head coil		12ch head coil
Pulse sequence		SE-EPI
Acceleration mode		ASSET or multiband
Acceleration factor		2
TR (ms)		12,000
TE (ms)		Minimum (61.6–81)
NEX/NSA		1
Bandwidth (kHz, Hz/Px, WFS(Px))		250 kHz
Matrix size (reconstruction)		256*256
Matrix size (acquisition)		128*128
FOV	FH (mm)	175
	AP (mm)	256
	RL (mm)	256
Voxel size	FH (mm)	2.5
	AP (mm)	2
	RL (mm)	2
Slice	Thickness (mm)	2.5
	Interslice spacing (mm)	0
	Number of slices	70
	Slice orientation	Axial
Phase encoding direction		AP
MPG type (monopolar/bipolar)		Monopolar
Number of MPG directions		30
b-values (s)		1,000
Number of T2 (b=0) acquisition		5
Shim (auto/manual)		Auto
Fat suppression (Yes/No)		Yes
Fat suppression mode (CHESS/SPIR/Water excitation)		CHESS
Scan duration		7:12

PRESS short TE

Head coil		12ch head coil
Pulse sequence		MRS Probe-Press
TR (ms)		2,000
TE (ms)		30
Voxel thickness (mm)		15
CSI slice thickness (mm)		30
Phase encoding direction		LR
FOV freq (cm)		24
Voxel size	FH (mm)	15
	AP (mm)	20
	RL (mm)	20
Total number of scans		64
NEX/NSA		2
Shim (auto/manual)		Auto
Fat suppression (Yes/No)		No
Fat suppression mode (CHESS/SPIR/Water excitation)		No
Scan duration		2:48

PRESS long TE

Head coil		12ch head coil
Pulse sequence		MRS Probe-Press
TR (ms)		2,000
TE (ms)		135
Voxel thickness (mm)		15
CSI slice thickness		30
Phase encoding direction		LR
FOV freq (cm)		24
Voxel size	FH (mm)	15
	AP (mm)	20
	RL (mm)	20
Total number of scans		64
NEX/NSA		2
Shim (auto/manual)		Auto
Fat suppression (Yes/No)		No
Fat suppression mode (CHESS/SPIR/Water excitation)		No
Scan duration		2:48

Synthetic MRI

Head coil		12ch head coil
Pulse sequence		2D MAGiC
Acceleration mode		ASSET
Acceleration factor		2
TR (ms)		7,000
Number of TEs		2
TE (ms)		15.2
Effective TE		91.2
Echo train length		16
Matrix size (reconstruction)		256*256
Matrix size (acquisition)		256*192
FOV	FH (ms)	160
	AP (ms)	240
	RL (ms)	192
Voxel size	FH (ms)	4
	AP (ms)	0.9
	RL (ms)	1.2
Slice	Thickness (mm)	4
	Interslice spacing (mm)	0
	Number of slices	42
	Slice orientation	Axial
Phase encoding direction		LR
Fat shift direction		Normal (A)
NEX/NSA		1
Bandwidth (kHz, Hz/Px, WFS(Px))		31.25 kHz
Shim (auto/manual)		Auto
Fat suppression (Yes/No)		No
Fat suppression mode (CHESS/SPIR/Water excitation)		No
Scan duration		5:08
